# A pan-European epidemiological study reveals honey bee colony survival depends on beekeeper education and disease control

**DOI:** 10.1371/journal.pone.0172591

**Published:** 2017-03-09

**Authors:** Antoine Jacques, Marion Laurent, EPILOBEE Consortium, Magali Ribière-Chabert, Mathilde Saussac, Stéphanie Bougeard, Giles E. Budge, Pascal Hendrikx, Marie-Pierre Chauzat

**Affiliations:** 1 Unit of coordination and support to surveillance, ANSES, Scientific Affairs Department for Laboratories, Maisons-Alfort, France; 2 Unit of Honey bee Pathology, ANSES, European Union and National Reference Laboratory for Honey bee Health, Sophia Antipolis, France; 3 Unit of Epidemiology and Welfare of Pork, ANSES, Ploufragan, France; 4 Fera, Sand Hutton, York, United Kingdom; 5 Institute for Agri-Food Research and Innovation, Newcastle University, Newcastle upon Tyne, Tyne and Wear, United Kingdom; Universidade de São paulo, BRAZIL

## Abstract

Reports of honey bee population decline has spurred many national efforts to understand the extent of the problem and to identify causative or associated factors. However, our collective understanding of the factors has been hampered by a lack of joined up trans-national effort. Moreover, the impacts of beekeeper knowledge and beekeeping management practices have often been overlooked, despite honey bees being a managed pollinator. Here, we established a standardised active monitoring network for 5 798 apiaries over two consecutive years to quantify honey bee colony mortality across 17 European countries. Our data demonstrate that overwinter losses ranged between 2% and 32%, and that high summer losses were likely to follow high winter losses. Multivariate Poisson regression models revealed that hobbyist beekeepers with small apiaries and little experience in beekeeping had double the winter mortality rate when compared to professional beekeepers. Furthermore, honey bees kept by professional beekeepers never showed signs of disease, unlike apiaries from hobbyist beekeepers that had symptoms of bacterial infection and heavy *Varroa* infestation. Our data highlight beekeeper background and apicultural practices as major drivers of honey bee colony losses. The benefits of conducting trans-national monitoring schemes and improving beekeeper training are discussed.

## Introduction

Honey bees are highly effective pollinators with an annual global contribution to crop productivity of € 147 million [1]. Recent decades have seen heightened concern about honey bee colony mortaility across the United States [2, 3], Asia [4] and Europe [5]. Whilst the global number of managed colonies has risen by about 45% over the last 60 years [6, 7], the seemingly unpredicable loss of honey bee colonies exacerbates the shortage of pollinators leading to concerns that pollination deficits may limit crop production [8]. Indeed in the US, it was shown that the quantity and quality of pollination services haved declined through time [9].

Honey bees are subject to many interacting pressures including pests, pathogens, pesticides and climate change—for a review see Vanbergen et al.[10]. National studies suggest different drivers of poor honey bee colony health depending on the geography. Pathogens were linked to poor honey bee health in the UK [11] and in Germany [12], pathogens and pesticides in Italy [13], while this was not found to be the case in Africa [14]. A first and recent Europe-wide experiment clearly indicated the presence of genotype-environment interactions originating from specific local adaptation of the honey bee populations [15]. Indeed, the most important result of this study was a significantly higher survivorship of the local genotypes compared to the non-local ones [16]. In the US, the diagnosed causes of overwintering mortality were different according to the beekeeper operation type: backyard beekeepers generally identified “manageable” factors (e.g., starvation, weak colony in the fall), while commercial beekeepers suggested environmental factors including pesticides [2]. In South-Africa, the effect of migratory beekeeping practices was found significant on colony losses, migratory beekeepers losing on average more colonies than stationary beekeepers [17]. For pollinators in general, other studies have shown the negative impact of land-use intensification [18], and interacting negative effects of human-induced changes on climate [19]. Despite all these studies, our collective understanding of honey bee health has been hampered by a lack of collaborative trans-national efforts following common protocols and the impacts of beekeeper knowledge and beekeeping management practices has often been overlooked, despite honey bees being a managed pollinator.

The objective of the present study was to identify the key risk factors surrounding honey bee colony mortality through data from the first surveillance programme based on randomly selected participants and deploying standardised methods to monitor honey bee colony health, pests, diseases and management practices across 17 European countries.

## Materials and methods

### Building a surveillance network

The selection of Member States taking part to EPILOBEE, the methodology to randomly pick up the apiaries and the beekeepers, the evaluation of the protocols set up in each Member State are described in details in Chauzat et al. [20] and in the guidelines published by the European Reference Laboratory for Honey bee Health (EURL) [21, 22].

The EPILOBEE protocol is described briefly below. Surveillance was implemented during two consecutive years, between autumn 2012 and summer 2014. Three visits were set up in each Member State: before winter (autumn visit: V1), after winter (spring visit: V2) and during the beekeeping season (summer visit: V3). At each visit, beekeeping practices and clinical signs of the main honey bee diseases were recorded during field inspections using a standardised questionnaire. If colonies exhibited clinical signs of a disease, samples were collected for subsequent laboratory analyses. The main honey bee diseases clinically investigated were those listed for notification for intra-EU trade and import rules or for national eradication programmes at the European level [23, 24]: the fungal disease Nosemosis; the parasitic disease varroosis; the two main bacterial diseases affecting honey bee brood; the American foulbrood (AFB) and the European foulbrood (EFB); and a viral disease caused by the chronic bee paralysis virus (CBPV). An apiary was considered to be suffering from a disease after its diagnosis in one or more colonies. All case definitions were agreed between the EURL and the Member States (20).

An agreed sampling protocol was circulated by the EURL after consultation with all participating Member States and is available upon request to the EURL [20]. Subsequently, each Member State organised training, managed the visits and stored the national data in an online database. For the second year, at least one third of the total beekeepers from the first year was renewed and for comparison the same methodology was utilised.

Population representativeness was achieved through random sampling of apiaries (primary units) and bee colonies (secondary units) in each Member State considered to be representative of the national beekeeping population [20]. England & Wales (taken as one Member State) did not take part in the second year of the program.

### Study population studied and retained variables

To calculate the colony mortality rates, only the apiaries with three consecutive visits and consistent data needed for mortality calculation were included. Data consistency was tested through several checks applied to the dataset including some editing to retain as much data as possible for analysis [25]. The consistency between various variables was checked; for exemple, the number of colonies randomly selected at the first visit and information reported at the second visit; the number of colonies randomly selected at the first visit and the size of the apiary; or the number of colonies owned by the beekeeper and the size of the apiary. After quality checking, 2 332 apiaries (out of 3 053) were retained for the analysis in the first year and 2 426 apiaries (out of 2 745) for the second year. Apiary was designated as the epidemiological unit.

Among the available 138 variables in 12 tables, 36 were selected for the statistical analysis after a Delphi-like selection. In summary, these 36 retained variables were related to the beekeeper (age, activity and experience in beekeeping), the operation type (type of production i.e. honey, pollen, queens), the operation scale (the number of colonies owned by the beekeeper, the number of colonies in the visited apiary), the husbandry (honey bee subspecies [26], swarms and queens produced or bought), the disease state, whether depopulation had been observed in the apiary (clinical signs and mortality of colonies or honey bees observed before and during site visits) and the landscape surrounding the apiary. They were used as explanatory variables with the seasonal and the winter mortalities as response variables. With the seasonal mortality as response variable, the previous winter mortality was included as a supplementary explanatory variable.

### Statistical methods

First, data sets from each year were considered independently and the 17 Member States were clustered according to honey bee colony mortality. Second, for each year considered again independently, the links between the seasonal and the winter mortalities were explored. To work with a complete dataset, missing data were completed using the data imputation method based on multiple correspondence analysis. Finally, risk factors of mortality were sought on the overall study including both years of data by means of generalised linear models (see below).

### Clustering the Member States according to yearly mortality

Yearly honey bee colony mortality was studied pooling together the winter mortality and the seasonal mortality on 2 332 apiaries for the first year and 2 426 apiaries for the second year. The 17 Member States were clustered according to mortality patterns (i.e. groups with high mortality rates *versus* lower mortality rates) using vectors composed of two values (for the winter mortality and for the seasonal mortality rates). The 17 vectors were subsequently analysed through the hierarchical clustering of observations on the main principal components function (HCPC) from the FactoMineR R package [27]. This resulted in producing groups of Member States with similar annual mortalities for the first and second year separately.

### Link between the winter and the seasonal mortalities

This specific analysis was performed for EPILOBEE first year and second year separately. Spearman correlation coefficients were calculated taking into account two variables for each year: a first one corresponding to winter mortality rates and a second one corresponding to seasonal mortality rates.

### Risk factors related to honey bee colony mortality (both years combined)

Considering the missing data in data sets from each year, 1 139 (out of 2 332) apiaries should have been kept in the analysis for the first year, and 1 020 (out of 2 426) for the second year. To take into acount a complete dataset, an imputation method was performed to process the missing data separately for each year, based on multiple correspondence analysis (MCA) [28] through the functions estim_ncpMCA and imputeMCA from the missMDA R package [29]. After imputation, data sets from each year were merged and the new global dataset was used in the following analysis.

The winter and seasonal honey bee colony mortalities (response variables) were considered as count numbers with quasi-Poisson distributions. In the subsequent analysis, the models used were the quasi-Poisson generalised linear models.

Calculations of p-values (associated to the likelihood ratio) were implemented for the winter mortality and the seasonal mortality for each explanatory variable (glm function R package). The 0.20 threshold was used to select the variables kept in the study: the ones with a p-value superior to the threshold were removed from the study, the others variables being incorporated in the subsequent steps.

To avoid multicollinearity in Poisson regressions, variables from each theme (e.g., diseases, management) were summarized in a single categorical latent variable through MCA and classification using the FactoMineR package [30] as detailed in Jacques et al. [25]. A unique new synthetic explanatory variable resulting from clustering and composed of seven categories was included in the generalised linear model as a fixed effect. The *Country* and the *year* variables were included as random effects. For each mortality, a mixed multivariate Poisson regression model was performed using the glmer function from the lme4 R package with a log link [31]. Thereafter, contrasts were used to compare mortalities of each cluster of the synthetic explanatory variable to the other ones.

At the end of the classification, the seven clusters of the synthetic variable were built according to eight criteria: the typology of the beekeeper (hobby, part time or professional), the size of the apiaries and of the colonies according to four classes: size 1 = operation having less than 50 colonies in total and apiary having less than 20 colonies; size 2 = operation having less than 50 colonies and apiary having between 21 and 50 colonies; size 3 = operation having between 51 and 300 colonies and apiary having more than 50 colonies; and size 4 = operation with more than 300 colonies and apiaries having more than 50 colonies. The age of the beekeeper was recorded in four groups (less than 30 years of age, between 30 and 45 years, between 45 and 65 years and more than 65 years of age). The involvement in a cooperative treatment against *Varroa* and the observation of clinical diseases in colonies visited and the subsequent confirmation in laboratory were also considered in the synthetic variable. The actions implemented at the operation level to improve the quality of production were reported under the section *Apiary management*: if the beekeeper produced his/her own queens and swarms or whether he/she bought some; if the management of the bee population by acquiring swarms and queens was intended to increase the production, to compensate the bad health conditions or to maintain the livestock; which subspecies of honey bees were used in the colonies visited; which environment surrounded the apiary under study according to the beekeeper; what kind of main production was targeted by the beekeeper; if the colonies were merged; and which honeyflow was targeted by the migration. The bee subspecies were reported by the beekeepers. There was no morphological nor molecular analysis conducted on honeybees to assess the subspecies. The environment surrounded the apiary under study was also reported by the beekeepers. No landscape measures were recorded to assess this criterion. The section on the *beekeeper background* gathered the experience in beekeeping (training, qualification, how long he/she is a beekeeper) and some practices (using a apiarist book, be part of a beekeeping organisation). The last section noted the presence of any health events in the apiary before the start of the project. For seasonal mortality only, a supplementary criteria was added to account for the winter colony mortality previously recorded.

## Results

### Clustering the Member States according to yearly mortality

**Fig 1:**
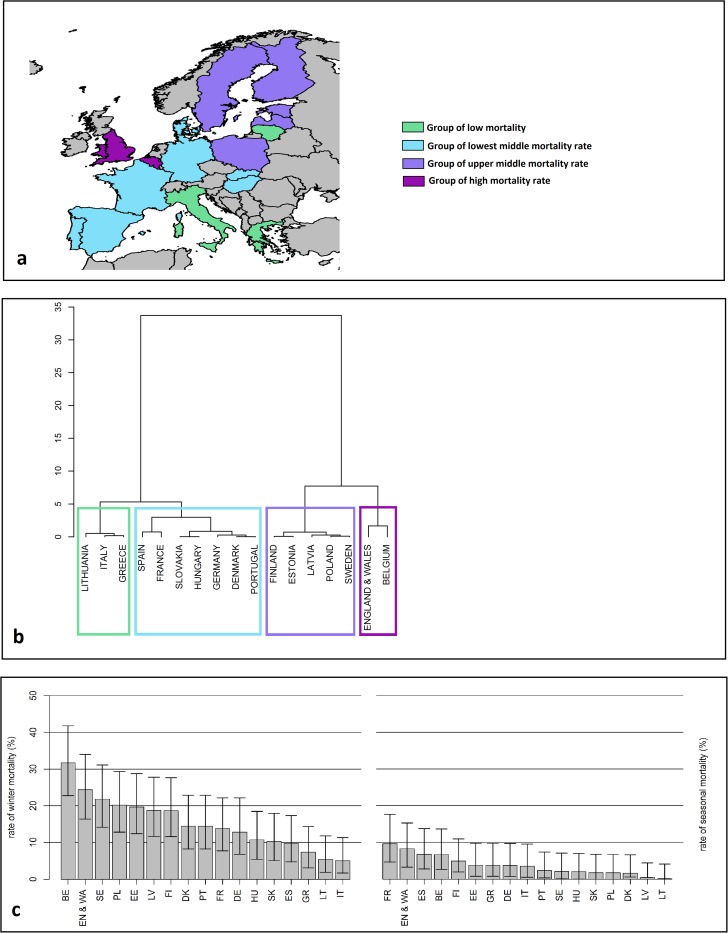
The Four Clusters or the Yearly Honey Bee Colony Mortality for EPILOBEE *first* year. They are **i**llustrated by a map (a) and a dendogram (b) and broken into the winter (c) and the seasonal (d) mortality rates. The vertical segments represent the 95% confidence intervals. BE = Belgium; DE = Germany; DK = Denmark; EE = Estonia; EN & WA = England & Wales; ES = Spain; FI = Finland; FR = France; GR = Greece; HU = Hungary; IT = Italy; LT = Lithuania; LV = Latvia; PL = Poland; PT = Portugal; SE = Sweden; SK = Slovakia.

During the first year, the Member States were clustered into four groups regarding the yearly mortality (Fig 1A and 1B): high mortality rates (Belgium and England & Wales), upper middle mortality rate (Estonia, Finland, Latvia, Poland and Sweden), lowest middle mortality rate (France, Denmark, Germany, Hungary, Portugal, Slovakia and Spain) and low winter mortality rate (Greece, Italy and Lithuania). When broken into winter and seasonal honey bee colony mortalities, the rates ranged from 31.73% (Belgium) to 5.01% (Italy) for winter mortality; and from 9.63% (France) to 0.09% (Lithuania) for seasonal honey bee colony mortalities (Fig 1C and 1D).

**Fig 2:**
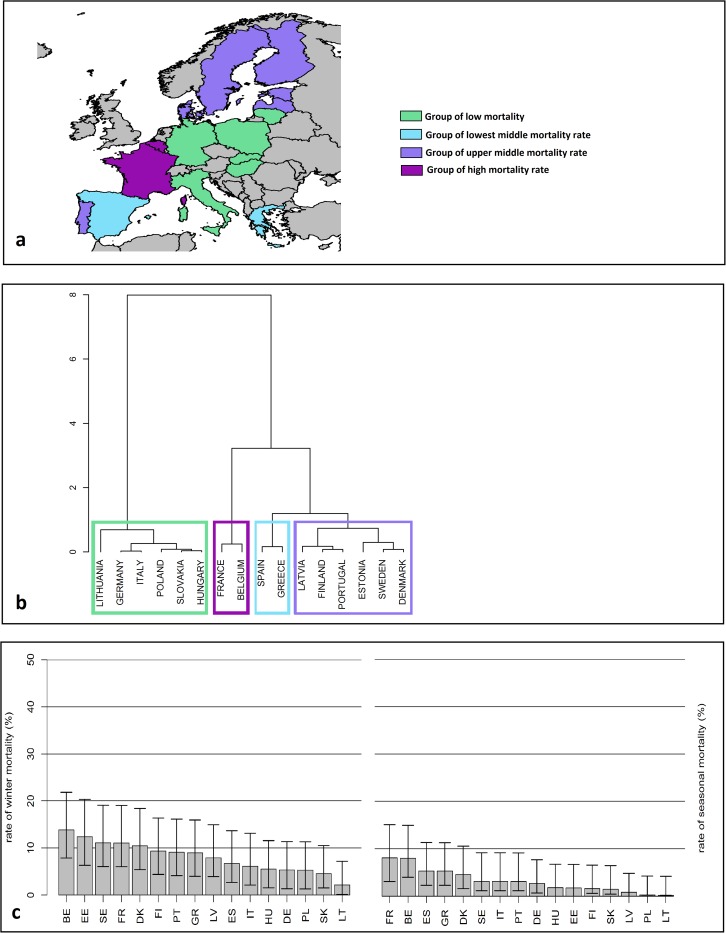
The Four Clusters or the Yearly Honey Bee Colony Mortality for EPILOBEE *second* year. They are illustrated by a map (a) and a dendogram (b) for EPILOBEE and broken into the winter (c) and the seasonal (d) mortality rates. The vertical segments represent the 95% confidence intervals. BE = Belgium; DE = Germany; DK = Denmark; EE = Estonia; ES = Spain; FI = Finland; FR = France; GR = Greece; HU = Hungary; IT = Italy; LT = Lithuania; LV = Latvia; PL = Poland; PT = Portugal; SE = Sweden; SK = Slovakia.

During the second year, four different groups were also produced with Belgium and France being part of the group with the higher mortality rates. Denmark, Estonia, Finland, Latvia, Portugal and Sweden were in the group with upper middle mortality rate. Greece and Spain were in the group of lowest middle mortality rate. The group with the lowest winter mortality rate included Germany, Hungary, Italy, Lithuania, Poland and Slovakia (Fig 2A and 2B). When broken into winter and seasonal mortalities, the rates ranged from 13.85% (Belgium) to 2.16% (Lithuania) for winter mortality and from 8.06% (France) to 0.16% (Lithuania) for the seasonal mortality (Fig 2C and 2D).

### Link between the winter and the seasonal mortalities

A positive though weak link was found between the winter and the seasonal mortalities of the first and the second year. The Spearman coefficients were statistically different from zero for both years (p-value = 6.10^−4^ and 2.10^−12^ respectively). The winter and the seasonal mortalities were positively linked meaning that in an apiary, when the mortality rate was strong over the winter, during the subsequent summer, the mortality rate had a tendency to be high. The Spearman correlation coefficient was a little stronger for the second year (0.142) compared to the first year (0.071).

### Risk factors related to honey bee colony mortality

After combining the complete dataset from both survey years, 33 variables were included in the analysis of the winter mortality and 28 for the seasonal mortality. The *year* effect was significant for both years of the study with a stronger effect on the winter mortality (p-value = 1.10^−37^) when compared to the seasonal mortality (p-value = 1.10^−3^).

The new synthetic explanatory variable generated from the clustering was divided into seven categories (W1 to W7 and S1 to S7, for winter and seasonal mortality respectively). Mortality rates were calculated with the multivariate Poisson regression models for each cluster and for each mortality: winter (Table 1) and seasonal (Table 2). For the winter mortality, hobby beekeepers with a small number of colonies (size 1) were part of four clusters (W1, W3 to W5). Clusters W6 and W7 gathering part time beekeepers and professionnals were only attached to the cluster W2 with a high number of colonies (size 3). Hobbyist beekeepers with small apiaries and little experience in beekeeping had double the winter mortality rate (14.04%—cluster W1) when compared to professional beekeepers (8.11%—cluster W2). Furthermore, honey bees kept by professional beekeepers never showed any signs of disease during the visits, unlike apiaries from hobbyist beekeepers that frequently had symptoms of heavy *Varroa* infestation (cluster W1). In the cluster with the highest winter mortality, the beekeepers were over 65 years of age. Their operation was located around an environment with farmland town or woods (Table 1). The apiaries aimed at producing queens. The beekeepers did not attend any beekeeping training during the past three years, did not register beekeeping management in an apiarist book, had no qualification. In this cluster, the beekeepers were not members of any beekeeping organisations and did not participate in any cooperative treatment against *Varroa*. They had an experience in beekeeping between two and five years. The apiaries suffered from varroosis at the autumn visit. In the cluster W2, with the lowest winter mortality (8,11%), beekeepers between 30 and 45 years of age had large apiaries and operations and were located around a floral environment (Table 1). The migration of apiaries targeted crops most of the time (or a diverse environment). The apiary management promoted the increase of the livestock (producing more than ten queens and swarms) and colonies were merged. The productions were diverse (honey, queens, pollen). The beekeepers attended a beekeeping training during the past three years, used an apiarist book, had a qualification in beekeeping, were members of a beekeeping organisation, and had an experience in beekeeping higher than five years. The apiaries did not suffer from any disease at the autumn visit.

**Table 1:** Honey bee colony winter mortalities calculated for the different clusters during EPILOBEE 2012–2014 with the features of each cluster.

Cluster (number of apiaries)		W1	W2	W3	W4	W5	W6	W7
		(n = 403)	(n = 695)	(n = 1324)	(n = 258)	(n = 710)	(n = 944)	(n = 424)
**Winter mortality (%)**	** **	**14.04**	**8.11**	**9.50**	**9.74**	**11.46**	**8.66**	**12.57**
**Typology**	** **	hobby	professional	hobby	Hobby	hobby	part time	part time
**Size (apiary & operation)**	** **	size 1	size 3	size 1	size 1	size 1	size 3	size 2
**Age of the beekeeper (years)**	** **	Over 65	30–45	Over 65	Less than 30	30–45	45–65	45–65
**Beekeeper background**	*Training*	*No*	Yes	Yes	No	No	Yes	Yes
	*Apiarist book*	*No*	Yes	Yes	No	No	Yes	Yes
	*Qualification*	*No*	Yes	Yes	No	No	Yes	Yes
	*Member of bkp org*	*No*	Yes	Yes	No	No	Yes	Yes
	*Bkp experience (years)*	*2 to 5*	> 5	> 5	< 2	2 to 5	> 5	> 5
**Cooperative treatment against *Varroa***	** **	No	No	Yes	NCOR	No	Yes	No
**Apiary management**	*Q & S production*	No information	More than 10	No information		No		More than 10
	*Q & S bought*	No information	No	No information		No		No
	*Management*	No information	Lvstk	No information		Production + HC + Lvstk		Lvstk
	*Subspecies*	Buckfast / Hybrid / *A*. *m*. *lig*	*A*. *m*. *iberiensis / A*. *m*. *ccm*	Buckfast / Hybrid / *A*. *m*. *lig*.		Local bees / *A*. *m*. *carnica*		*A*. *m*. *iberiensis /A*. *m*. *ccm*
	*Environment*	Farmland / Town / Wood	Floral	Farmland / Town / Wood		Diverse		Floral
	*Production*	Queens	Diverse	Queens		Honey		Diverse
	*Colonies merged*	NCOR	Yes	NCOR		No		Yes
	*Migration*	No information	Crops & Diverse	No information		No information		Crops & Diverse
**Clinical disease observed**	** **	Varroosis	No clinical disease observed	No clinical disease observed	NCOR	No clinical disease observed	No clinical disease observed	AFB & Varrosis
**Health events**	** **	No	No	Yes	NCOR	Yes	Yes	No

The mortality rates of all the clusters were significantly different from each other (p<0.05) except between the clusters W3 and W4 (p = 0.18). Q & S = queens and swarms; HC = Health Conditions; Lvstk = Livestock; A. m. = *Apis melifera*; lig. = *ligustica*; ccm = *carpatica*, *caucasia or macedonia*; bkp = beekeeper; org = organisation; size 1 = operation ≤50 colonies and apiary ≤ 20 colonies; size 2 = operation ≤50 and apiary between 21 and 50; size 3 = operation between 51 and 300 and apiary >50; Diverse environment = apiary surroundings composed of two or more different types of environment; Farmland/Town/Wood environment = apiary surroundings either composed of only farmland or only town or only wood environment. Diverse production = honey, queens and pollen. Diverse migration = migration targeted locations mixing crops and wildflowers. NCOR = No Category Over Represented, i.e. for a given variable and a given cluster, among all the categories, none was over represented. No information = no information provided for these apiaries. For further details on clusters and categories, please refer to the text.

**Table 2:** Honey bee colony seasonal mortalities calculated for the different clusters during EPILOBEE 2012–2014 with the features of each cluster.

Cluster (number of apiaries)		S1	S2	S3	S4	S5	S6	S7
		(n = 103)	(n = 1299)	(n = 633)	(n = 794)	(n = 684)	(n = 885)	(n = 360)
**Seasonal mortality (%)**	** **	**7.81**	**1.81**	**3.40**	**2.48**	**2.00**	**3.98**	**2.04**
**Typology**	** **	hobby	hobby	hobby	hobby	part time	part time	professional
**Size (apiary & operation)**	** **	NCOR	size 1	size 1	size 1	size 2	size 2	size 3
**Age of the beekeeper (years)**	** **	NCOR	45–65	Over 65	Over 65	45–65	30–45	30–45
**Beekeeper background**	*Training*	No	Yes	No	No	Yes	Yes	Yes
	*Apiarist book*	No	Yes	No	Yes	Yes	No	Yes
**Cooperative treatment against *Varroa***	** **	No	Yes	No	No	Yes	No	No
**Apiary management**	*Q & S production*	NCOR	No information	No information	No	More than 10		
	*Q & S bought*		No information	No information	No	More than 10 Q & 5 S		
	*Management*		No information	No information	Lvstk	Production + HC + Livestock		
	*Subspecies*		Buckfast / Hybrid */ A*. *m*. *lig*.	Buckfast / Hybrid / *A*. *m*. *lig*.	*Local bees / A*. *m*. *iberiensis*	*A*. *m*. *carnica / A*. *m*. *ccm*		
	*Colonies merged*		NCOR	NCOR	No	Yes		
**Clinical disease observed**	** **	AFB	No clinical disease observed	Varrosis	No clinical disease observed	NCOR	Varrosis	No clinical disease observed
**Health events**	** **	No	Yes	No	No	Yes	No	No
**Previous winter mortality (%)**	** **	21–50	No mortality	21–50	No mortality	6–10	1–5	6–10

The mortality rates of all the clusters were significantly different from each other (p<0.05) except between the clusters S5 and S7 (p = 0.48). Q & S = queens and swarms; HC = Health Conditions; Lvstk = Livestock; A. m. = *Apis melifera*; ccm = *carpatica*, *caucasia or macedonia*; lig. = *ligustica*; bkp = beekeeper; org = organisation; size 1 = operation ≤50 colonies and apiary ≤ 20 colonies; size 2 = operation ≤50 and apiary between 21 and 50; size 3 = operation between 51 and 300 and apiary >50. NCOR: No Category Over Represented, i.e. for a given variable and a given cluster, among all the categories, none was over represented. No information = no information provided for these apiaries. For further details on clusters and categories, please refer to the text.

During the season (Table 2), the highest colony mortality rate (7.81%) was calculated for the cluster S1. In the same way as for the winter mortality, the beekeepers from this cluster were distinguished by their lack of attendance to any beekeeping training during the past three years. The beekeepers were not part of any cooperative treatment against *Varroa* before the onset of the visits and did not use any apiarist book. During the previous winter, the colony mortality rates in these apiaries were between 21% and 50%. The beekeepers stated that they did not experience any health events before commencing the study but cases of AFB were found in the apiaries during the spring visit. The lowest seasonal mortality (1.81%—cluster S2) was attributed to the cluster with beekeepers who attended a beekeeping training and used an apiarist book (Table 2). These beekeepers took part to a cooperative treatment against *Varroa*. These apiaries did not experience any mortality during the previous winter. Regarding diseases, the apiaries experienced some health events before the study but did not suffer from any disease at the spring visit.

## Discussion

Our results demonstrate that overwinter losses ranged between 2% and 32%, and that high summer losses were likely to follow high winter losses. Multivariate Poisson regression models revealed that hobbyist beekeepers with small apiaries and little experience in beekeeping had double the winter mortality rate when compared to professional beekeepers. Furthermore, honey bees kept by professional beekeepers never showed signs of disease during the visits, unlike apiaries from hobbyist beekeepers that had often symptoms of bacterial infection and/or heavy *Varroa* infestation observed during the visits. Our results show that the main factors protecting honey bee colonies are beekeeper background and practices. More efforts are needed in beekeeper training to promote good beekeeping practices and achieve early identification of clinical signs of disease. During the first year of the study there was no clear geographical pattern for the distribution of yearly mortality rates (including both winter and seasonal rates). Conversely, during the second year, the Member States with similar mortality rates were distributed according to geographical patterns, grouped in patches. The winter 2012–2013 was particularly long and cold across Europe. This was not the case for the following winter (2013–2014) which was rather mild [32]. Mild winters are known to favour the build-up of Varroa mite populations. According to a longitudinal study run in Europe, the colony location strongly influence autumn mite loads and viral prevalence [15]. Multi-year longitudinal monitoring of colony health can identify region-specific risk factors associated with colony mortality [33]. Once these factors have been identified, they can inform on management and research priorities including pesticide uses [33]. It has been recently suggested that different honey bee populations may have developed their own specific resistance mechanisms tailored to match the challenge of the environment they are located in, including the locally prevailing combination of pathogens and pathogen variants [16]. Similar results were shown in England and Wales for AFB exhibiting significant spatial aggregation at distances from 10 to 30 km [34]. In the field of ecotoxicology, sublethal effects of neonicotinoid pesticides were also modified in magnitude by environmental interactions specific to the landscape and time of exposure events [35]. These observations advocate the use of honey bee health surveys to be conducted at the landscape scale, and to include environmental factors. However, the distribution of the Member States with similar mortalities observed during the first year of the study without any geographical pattern may indicate that weather could be an overwhelming factor that can mask regional effects. In the US data showed that beekeepers in northern states lose more colonies than those in southern states [2, 33]. Therefore the role of climate in winter colony mortality could be further elucidated by repeating the surveys over serveral years. The mortality rates reported in EPILOBEE were relatively low compared to rates reported in the US which are consistenly around one-third for each winter [3]. It is not known whether these discrepancies could be attributed to sampling issues, analysis, biological pressures, landscapes or management factors, or any combination of the above. Commercial beekeeper in the US typically transport their hives thousands of miles per year, which is exceedingly rare in Europe. Although some studies have already highlighted the stress induced by these transportations, how these factors influence the various mortality rates remains largely unknown at colony level [36, 37].

We also present the first evidence of a relationship between winter and seasonal mortalities. Although winter colony mortality is commonly used to quantify losses in a standardized way, the colony mortality during a full year should also be taken into consideration to estimate population level trends. Colony losses are highly dependant on beekeeper management which includes how colonies are taken care (disease detection) but also which honey flow is targeted and therefore potential specific pesticide exposure [38]. Thus, future surveillance studies should encompass information on land use and pesticide analyses.

The statistical analysis of our data from the first year has shown that size of the operation, apiary and the clinical disease (varroosis, AFB and nosemosis) observed during the previous autumn were significant risk indicators of overwinter honey bee colony survival [20]. In the present study, our findings find a similar result when considering two study years. The study variables were grouped into seven summary clusters. The clusters with the highest and the lowest mortality were distinguished by occurrence of diseases and beekeeper background. When the data set was extended to include both years, only prior observations of varroosis for the winter mortality and AFB for the summer mortality were found to be significant risk factors. Beekeepers with better knowledge on disease detection and management (specifically for varroosis and AFB), applying earlier prophylactic measures and good beekeeping practices (e.g. preparation of colonies for winter) had lower mortality rates than others (Tables 1 & 2).

Recently, historical data have been used to explore the link between honey bee colony mortality and other risk factors such as changes in the political and socioeconomic systems [39]. Using FAO data, it was shown that the most dramatic decline in the number of colonies in Europe was statistically associated with the collapse of the socialistic regimes in the Eastern European countries after 1989. In contrast, the arrival of the parasitic mite *V*. *destructor* in the early eighties in Europe had no detectable effects on the number of managed colonies in the full European data set. This does not show that *Varroa* does not kill honey bee colonies. However, it shows how beekeepers efficiently adapt their operation to comply with the challenges set by pests even as lethal as *V*. *destructor* [39]. Our results are in accordance with the latter study showing that beekeepers tend to have practices that compensate for the colony losses. Indeed, during both years of the study, the number of swarms bought was positively linked with both mortalities (the more swarms were bought, the higher the colony mortality–results not shown). The statistical link was also significant between merged colonies and colony losses. This might reflect the additional work needed by a beekeeper to overcome colony losses: beekeepers got new swarms and merged colonies to maintain their livestock at an acceptable level in quantity and quality. In future studies, the assessment of beekeeper additional work to compensate for losses or weakness of colonies should be better quantified.

The limits of our descriptive epidemiological protocol should be taken into account when drawing conclusions. Indeed, any hypotheses expressed in this paper should be fully studied in dedicated experimental protocols to confirm the risk factors and clarify any potential causality [33]. Our study offers observational evidence to suggest the importance of beekeeper training and education. These results must be seen as preliminary until confirmed by direct experimental means.

## Conclusion

Our results show that the main factors protecting honey bee colonies are beekeeper background and practices. More efforts are needed in beekeeper training to promote good beekeeping practices and achieve early identification of clinical signs of disease. Considerable variation of colony losses exist across different Member States and between years. Climate conditions might have a strong effect on colony mortality during the whole year, requiring long term surveillance study to overcome the weather factor. Data from descriptive survey such as EPILOBEE should be used to set up dedicated protocols to study further targeted hypotheses. The promotion of regional scale studies of local practices should be encouraged. Further to this work, the causes of colony losses should be investigated by conducting studies on specific issues as potential causes of honey bee losses, for example case-control studies that include pesticide analyses and landscape recording.
